# Co-delivery of paclitaxel and curcumin to foliate positive cancer cells using Pluronic-coated iron oxide nanoparticles

**DOI:** 10.1007/s40204-019-0118-5

**Published:** 2019-06-13

**Authors:** Chinmay G. Hiremath, Geetha B. Heggnnavar, Mahadevappa Y. Kariduraganavar, Murigendra B. Hiremath

**Affiliations:** 1 Department of Biotechnology and Microbiology, Karnatak University, Pavate Nagar, Dharwad, Karnataka 580003 India; 2 Department of Chemistry, Karnatak University, Dharwad, Karnataka 580003 India

**Keywords:** Pluronic, Magnetic nanoparticles, Folic acid, Curcumin, Paclitaxel

## Abstract

**Abstract:**

Active targeting of folic acid and passive targeting of magnetic nanoparticles to bring about co-delivery of hydrophobic chemotherapeutic agents were the focus of this work. Co-precipitation in alkaline environment was employed for synthesizing Fe_3_O_4_ nanoparticles and stabilized by oleic acid. Aqueous dispersibility of oleic acid coated nanoparticles was brought about by folic acid modified Pluronic F127 and Pluronic F127 mixture. Folic acid is used as a targeting agent which was joined to Pluronic F127 via diethylene glycol bis(3-aminopropyl) ether spacer. The nanocomposite was used to delivery hydrophobic anticancer drugs, paclitaxel, and curcumin. Successful modification at each step was confirmed by FTIR and NMR. Quantitative analysis of attached folic acid indicated a total of 84.34% amount of conjugation. Nanoparticles characterization revealed the hydrodynamic size of and nanocomposite to be 94.2 nm nanometres. Furthermore, transmission electron micrograph reveals the size of the nanoparticle to be 12.5 nm hence also shows the superparamagnetic activity. Drug encapsulation efficiency of 34.7% and 59.5% was noted for paclitaxel and curcumin, respectively. Cytotoxic property of drug-loaded nanocomposites was increased in case of folic acid functionalized nanoparticles and further increased in the presence of an external magnetic field. Cellular uptake increased in the folic acid conjugated sample. Further many folds in the presence of an external magnetic field.

**Graphic abstract:**

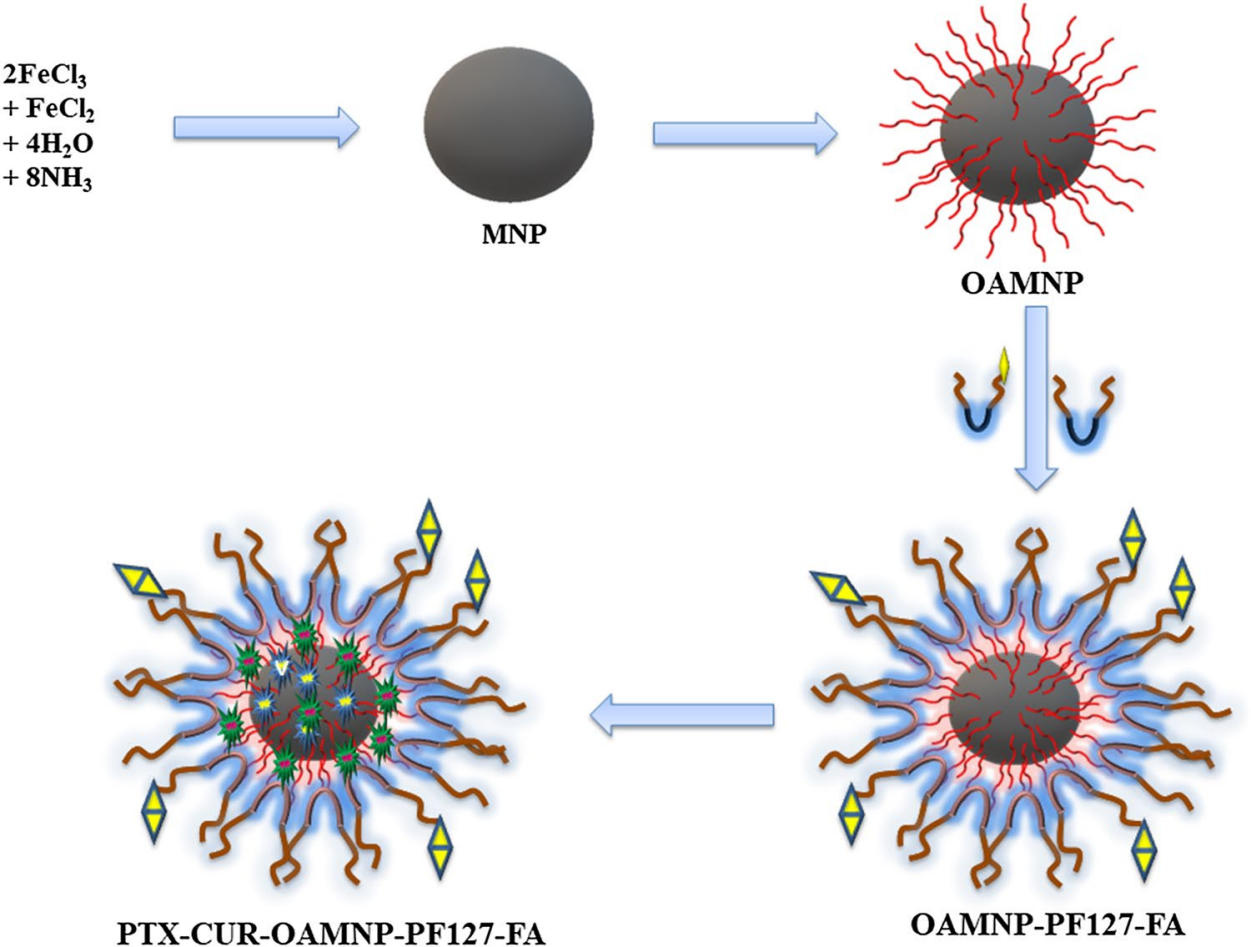

**Electronic supplementary material:**

The online version of this article (10.1007/s40204-019-0118-5) contains supplementary material, which is available to authorized users.

## Introduction

Cancer is one of the world’s highly distressing diseases, with 10 million new reports annually (Hamzehalipour Almaki et al. 2017). Chemotherapy drugs are effective in curbing cancer; however, drugs elicit toxicity. Toxicity is due to indiscriminate biodistribution thus drastically damaging patient’s quality of life. Moreover, some of the potent cancer chemotherapeutic agents suffer from low aqueous solubility and poor bioavailability (Brigger et al. 2002; Khodabandehloo et al. 2016). Targeted drug delivery, a promising and growing technology in cancer therapy, involves delivery of chemotherapeutic molecules directly to the site of cancer thus ensuring restricted drug distribution, enhancement of efficacy and safety of drug (Chauhan and Jain 2013). One such technology gaining importance is nanotechnology-based targeted drug delivery, which allows encapsulating the chemotherapeutic agents and also brings about site-specific distribution with a help cancer-specific or a tumor abundant markers for targeting cancer (Veiseh et al. 2010; Lomis et al. 2016).

There are a wide range of nanoparticulate materials, such as liposomes, polymeric nanoparticles, solid lipid, dendrimers, etc. being developed for the delivery of therapeutic compounds (Mudshinge et al. 2011). Fe_3_O_4_, magnetite, and nanoparticles are finding wide-ranging use in biomedical application, as it can be localized to required site of action using exterior magnetic field(Yallapu et al. 2011). Fe_3_O_4_ nanoparticle bestows good biocompatibility and post metabolism is converted into iron salts which is eventually incorporated by the body (Gupta and Gupta 2005; Sun et al. 2008; Veiseh et al. 2010). Often, capping agents are used to stabilise the nanoparticles, and one such capping agent is oleic acid (OA). OA is a biomolecule and is pharmaceutically inert material (Jin et al. 2010).

Pluronic F127 (PF127) is an A–B–A-type triblock polymer consisting of poly(ethylene oxide)—poly(propylene oxide)—poly(ethylene oxide) blocks, (PEO_100_–PPO_65_–PEO_100_). Pluronics display a twin property in water, PEO displays solubility in aquatic environment, and the PPO shows no aqueous solubility (Wang et al. 2007; Lin et al. 2009). The exterior PEO wreath inhibits protein interaction and stealth’s from immune recognition (Huang et al. 2009). Interior PPO block interacts hydrophobically with hydrophobic chemotherapeutic agents, i.e., it can encapsulate the drug molecule (Lin et al. 2009). Another advantage of PF127 is terminal hydroxyl groups which can be modified to desired functionality (Song et al. 2010).

Folic acid (FA) is vitamin B9, an essential biomolecule for the one-carbon metabolic reactions, methylation, DNA biosynthesis, and repair, and thus, there is amplified expression of folic acid receptors (FRs) in the breast, ovarian, endometrial, and colon cancer (Sudimack and Lee 2000; Sun et al. 2006; Locasale 2013). Conversely, the expression of FRs in typical human tissues is limited to a low level (Kelemen 2006). FA and FA conjugates undergo receptor-mediated endocytosis upon binding to FRs, so the FA-modified drug-delivery systems deliver desired therapeutics to tumor cells (Zhao et al. 2008; Cheung et al. 2016). Thus, hypothesizing enhanced potency and reduced off-site toxicity foliate-linked therapeutic agents in contrast to non-targeted drugs (Xia and Low 2010).

Paclitaxel is a potent microtubule inhibitor extracted from *Taxis brevifolia* (ten Tije et al. 2003). This highly potent molecule, due to its greasy nature, is subjected to poor bioavailability (Zähres et al. 2006). Reports of paclitaxel treatment in breast cancer indicate the condition of myelotoxicity and neurotoxicity in patients (Zähres et al. 2006; Pace et al. 2007). Furthermore, paclitaxel treatment, like other chemotherapeutic agents, exhibits chemoresistance, one of the major challenge, in cancer therapy, owing to ATP binding cassettes (ABC) and *p*-glycoprotein (PGP) transporters (Koutras et al. 2008; Gottesman et al. 2002). Furthermore, evidence suggests the involvement of nuclear factor κB (NF-κB), which promotes cell proliferation and up-regulates metastasis genes (Sui et al. 2013). Despite high potency to cure cancer, paclitaxel finds limited clinical use due to poor bioavailability, systemic toxicity, and drug resistance.

Curcumin is a pharmaceutically safe pleiotropic drug isolated from root modifications of *Curcuma longa* (Shishodia et al. 2005; Kang et al. 2009). Along with its anticancer property, curcumin suppresses the nuclear factor κB (NF-κB), a factor which up-regulates the proliferation and metastasis genes, thus curbs down chemoresistance induced by paclitaxel in breast cancer (Koutras et al. 2008; Hatcher et al. 2008; Kang et al. 2009). Furthermore, curcumin also down-regulates the expression of major ABC transporters which are major pathways in multidrug resistance in taxanes (Kuttan et al. 1985; Huang et al. 1988; Ma and Mumper 2013; Tian et al. 2016). Thus, a number of researchers think it is advisable to co-deliver paclitaxel with curcumin, since the latter is reported to bring about chemosensitization of former (Longley and Johnston 2005; Ganta and Amiji 2009; Yallapu et al. 2012; Sui et al. 2013).

Present work aimsto develop OA-coated iron oxide nanoparticle stabilized by FA-modified PF127 (OAMNPPF127FA) for active and passive delivery of hydrophobic drugs, curcumin and paclitaxel, to breast cancer cells. Folic acid was attached to PF127 by amine homofunctionalized Diethylene glycol moiety. We hypothesize hydrophobic drugs partition between OA and PPO hydrophobic corona. Furthermore, nanocomposites were considered to be thought externally localized and selectively target the foliate positive cell lines.

## Materials and methods

### Materials

Iron (III) chloride (FeCl_3_) and iron (II) chloride tetrahydrate (FeCl_2_ 4H_2_O) as nanoparticle components, ammonium hydroxide (30–33%M) for precipitation, Pluronic-F127, paclitaxel, folic acid, (1-ethyl-3-(3-dimethylaminopropyl) carbodiimide (EDC), and *N*-hydroxy succinamide (NHS) for crosslinking were procured from Sigma-Aldrich. Oleic acid, diethylene glycol bis (3-aminopropyl) ether, 1,1′-carbonyldiimidazole (CDI), and curcumin were purchased from TCI chemicals. 5-fluorouracil and dimethyl sulfoxide (DMSO) were purchased from MOLchem. Nitrogen purged Milli-Q was used in all the steps involved in the synthesis and formulation of magnetic nanoparticles.

### Synthesis of Pluronic P 127-CDI adduct

PF127-CDI adduct was synthesized as mentioned in publications (Fujita et al. 1999; Zhang et al. 2011). Purification of PF127 was brought about by dissolving 15 g of PF127 30 mL of acetone trailed by precipitation in chilled hexane. Thus, obtained precipitate was lyophilized and stored at room temperature. Furthermore, 2 g of CDI was dissolved in 15 mL of anhydrous DMSO, 10 g of purified PF127 was dissolved in 15 mL of anhydrous DMSO was added dropwise to above solution over 30 min under constant stirring. The mixture was permitted to stir for 12 h. The resultant solution was dispensed into an excess of cold diethyl ether to precipitate PF127-CDI adduct and to remove unreacted CDI. This process was repeated several times to remove unreacted CDI and the sample was vacuum dried and collected as a white powder.

### Synthesis of amino-terminated Pluronic F127 (PF127-NH_2_)

Solution of 12.7 g of PF127-CDI adduct dissolved in anhydrous DMSO was added dropwise to excess of diethylene glycol bis(3-aminopropyl)ether at ambient temperature over 30 min. The solution was stirred for 24 h. Purification of diethylene glycol bis(3-aminopropyl) ether modified PF127 (PF127-NH_2_) was brought about by dispensing solution in excess of chilled diethyl ether. Precipitation step was repeated several times to eliminate unreacted diethylene glycol bis(3 aminopropyl)ether. The sample was further purified by dialysis using 6 KDa membrane against water (Batrakova et al. 2005).

### Synthesis of foliate-conjugated Pluronic F127 (PF127-FA)

PF127FA was synthesized using EDC and NHS zero length cross linkers (Saul et al. 2003). Briefly, PF127-NH_2_ (500 mg, 0.079 mM), folic acid (105 mg, 0.237 mM), NHS (60 mg, 0.522 mM), and EDC (108 mg, 0.522 mM)were dissolved in 10 mL of DMSO. The mixture was stirred in an inert atmosphere in dark at room temperature for 24 h. The reaction mixture was diluted with water and dialysed against distilled water for 2 days.

### Synthesis of nanocomposites

Aqueous solutions of 0.1 M Fe(III) (100 mL) and 0.1 M Fe(II) (150 mL) were mixed and heated to 80 °C, stirred at 3000 rpm under an inert atmosphere. Furthermore, dropwise addition of 30 mLof ammonia (5 M) was brought about, over 3 min. To this reaction mixture, 300 µL of oleic acid was added and allowed to stir for 20 min to obtain oleic acid coated magnetic nanoparticles (OAMNP). The product was washed by magnetic decantation and lyophilized.

PF127 and PF127FA, in 9:1 ratio, were dissolved in water (making total weight of 50 mg). To PF127 solution, 10 mg of OAMNP suspended in chloroform was added. The components were sonicated to obtain OAMNPPF127FA. The particles obtained were washed 3 times using ultracentrifugation (30,000 rpm for 20 min at 10 °C) with nitrogen purged milli-Q water.

### Nanoparticles physical and chemical characterisation

Fourier transform infrared spectroscopy (FTIR) was recorded by Thermo Nicolet 8700, USA. Tiny amount of a sample was mixed with KBr to make a pellet. Samples were scanned in the range of 4000–500/cm.

H nuclear magnetic analysis of PF127-NH_2_ and PF127FA was conducted by dissolving sample in DMSO-d_6_ and measured using JEOL JNM-ECZ400S, USA. The analysis was performed with help of Delta 5.0.5 software.

UV–visible spectrophotometric analysis was performed to confirm conjugation and quantitatively measure FA content. A known quantity FA was dissolved in DMSO and diluted to different ratios to generate standard graph at 360 nm using Hitachi U-2900 spectrophotometer.

The hydrodynamic size and zeta potential of nanocomposites were scanned by dispersing 1 mg of the sample in 10 mL of Milli-Q water. The hydrodynamic size was measured at 25 °C using polystyrene zeta-potential cuvette. Zeta-potential analysis was carried out by injecting sample into graphite electrode zeta-potential cuvette. Particle size and zeta-potential analysis were conducted with Horiba, SZ-100.

A droplet of OAMNPPF172FA nanoparticles (an aqueous dispersion) was placed on a carbon-coated copper TEM network and was permitted to air dry. Particles were imaged using a Philips 201 TEM. The NIH ImageJ software was used to calculate the average particle size from the TEM photomicrograph. Sizes of 20 particles were measured to calculate the average particle diameter.

The XRD analysis of OAMNPPF172FA powder was carried out using a Bruker AXS D8 Advance. The parameters chosen for the measurement were 2*θ* steps of 0.02°, 6 s, and 2*θ* range from 20° to 80^o.^

Thermogravimetric analysis (TGA) was performed for pure OA, MNP, OAMNP, and OAMNPPF127FA by heating 600 °C at a ramp rate of 10 °C/min under nitrogen atmosphere (SDT Q600, USA.).

The magnetization and demagnetization profiles of OAMNP and OAMNPPF127FA were measured at 25 °C at 15 kOe applied magnetic field using, Lakeshore VSM 7410, vibrating sample magnetometer (VSM).

### Drug entrapment efficiency and in-vitro drug release

The engineered nanocomposites were exploited as a carrier of hydrophobic chemotherapeutic agents PTX and CUR. The loading of PTX and CUR in nanocomposites was done as reported previously (Jain et al. 2008). 50 mg of the OAMNPPF127FA was dispersed in 25 mL of Milli-Q water and was sonicated for 2 min. Solution 5 mg of PTX and 5 mg of CUR was dissolved in 5 mL of DCM was added dropwise to OAMNPPF127FA dispersion over 3 h. The solution was allowed to stir for 12 h at room temperature in dark. This allows the partitioning of the drug into the hydrophobic regions of OA and PPO moiety of PF127 surrounding the Fe_3_O_4_ nanoparticles. The un-partitioned drug was removed by centrifugation at 30,000 rpm (Beckman Coulter LT optima 60) for 30 min at 10 °C. The pellets of PTX and CUR loaded OAMNPPF127FA (PTX-CUR-OAMNPPF127FA) resuspended in a minimum quantity of water was lyophilized and stored at 4 °C.

The encapsulation efficiency (EE) and loading content (LC) of PTX and CUR in OAMNPP127FA were determined using Eqs. (1) and (2):

1 $${\text{DL \% }}\; = \;\frac{\text{Weight of drug in NP}}{\text{Weight of nanoparticle}}\; \times \;100,$$

2 $${\text{EE \% }}\; = \;\frac{\text{Weight of drug in NP}}{\text{Weight of drug fed}}\; \times \;100.$$

The drug release study was performed by centrifugation method (Wallace et al. 2012). 1 mg/mL of sample was suspended in PBS pH 7.4 with 0.5% tween 80. At specific time intervals, sample was centrifuged at 30000 rpm for 30 min and the supernatant was analyzed for drug content using UV–Vis spectrophotometer at 200 nm and 421 nm for paclitaxel and curcumin, respectively.

### Hemolysis assay

A biomaterial with intravenous route of administration should be considered for compatibility with blood. To assess the hemocompatibility of nanocomposites, hemolysis assay was performed (Slowing et al. 2009). To assess hemocompatibility, spectrophotometer-based hemolysis assay was performed. The blood sample was collected from a healthy donor in an EDTA treated vial. Erythrocytes harvested by centrifugation at 2000 rpm (Eppendorf, 5804R) for 5 min at 4 °C. The pellet was gently washed multiple times with PBS at pH 7.4 by centrifugation and resuspended in the PBS to make 4% erythrocytes concentration. 1 mL of erythrocytes suspension was mixed with 1 mL of nanoparticles (25–400 µg) and incubated for 1 h at 37 °C under gentle agitation. After incubation, the cells were centrifuged to settle the blood cells. The supernatant was analyzed for released hemoglobin photometric analysis (Hitachi, U-2900) at 540 nm. 1 mL of erythrocytes and 1 mL of PBS was used as a control for zero hemolysis. 1 mL of erythrocyte and 1 mL of PBS with 1% Triton X was used as 100% hemolysis. The erythrocyte lysis percentage was calculated according to the following equation:

3 $${\text{Percentage hemolysis}}\; = \;\frac{{{\text{Absorbance}}_{\text{Sample}} - {\text{Absorbance}}_{\text{Blank}} }}{{{\text{Absorbance}}_{{ 1 0 0 {\text{\% Lysis}}}} - {\text{Absorbance}}_{\text{Blank}} }}\; \times \; 100.$$

### In-vitro cytotoxicity assay

To assess the cytotoxic effects of Blank OAMNPPF127FA, PTX-CUR-OAMNPPF127, PTX-CUR-OAMNPPF127FA, and PTX-CUR-OAMNPPF127FA nanocomposites under magnetic field was determined using 3-[4,5-dimethylthiazol-2-yl]-2,5-diphenyl tetrazolium bromide (MTT) cell viability assay. 96 well assay plates were seeded with MCF-7 cells at a density of 5000 cells per well. After 24 h, the cells were treated with 0.01, 0.1, 1, 10, and 100 μg/mL concentrations of PTX-CUR, Blank OAMNPPF127FA, PTX-CUR-OAMNPPF127, and PTX-CUR-OAMNPPF127FA. To study the cell viability of PTX-CUR-OAMNPPF127FA under the magnetic field, cells were grown under 0.05 T magnet positioned under wells. After incubating for 48 h, the viability of cell was determined by adding dye solution (50 µl) to each well. Plates were incubated for 4 h at 37 °C in 5% CO_2_ atmosphere. Purple formazan crystals were solubilized in 100 µl of DMSO. Furthermore, absorbance was noted at 540 nm.

### Cellular uptake

Uptake of nanocomposites was determined by quantifying iron content in the cell by means of atomic absorption spectra (AAS) (Prijic et al. 2010; Dinda et al. 2012). MCF-7 were seeded in 6-well plates, at 7.2 × 10^6^ cells per well. Upon attainment confluence, growth media were swapped with 2 mL of OAMNPPF127 and OAMNPPF127FA suspension culture medium with (iron content of 200 µg/mL). Plates were incubated at 37 °C and 5% CO_2_ for duration of 60 and 240 min. Post-incubation cells were trypsinized and washed with DPBS to make sure thorough removal of media and remaining nanoparticles. Cells were collected by centrifugation and digested with 1 mL of HCl. After digestion, a 1:4 dilution in HCl (0.1 M) was measured for iron content by AAS.

### Statistics

SPSS 20 was used to statistically analyzed for Standard mean and standard deviation for MTT assay, drug release study, and cellular uptake.

## Results

### Synthesis OAMNPP127FA nanocomposites

**Fig. 1:**
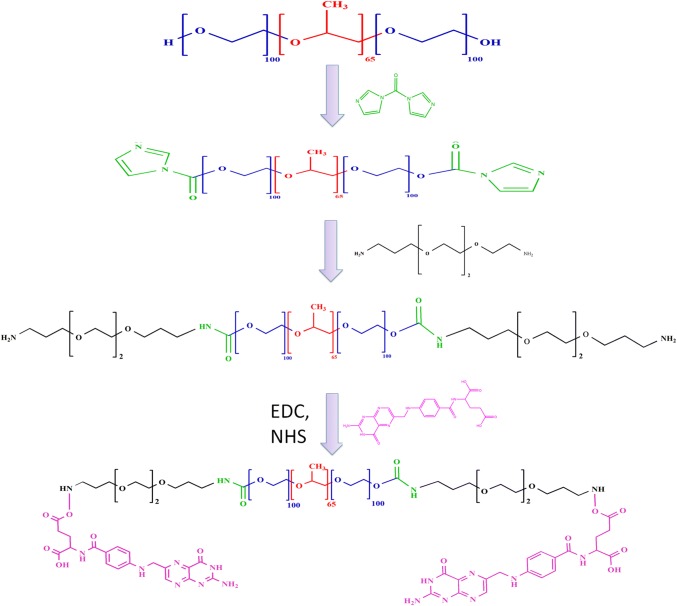
Schematic representation of PF127FA synthesis

FA-modified PF127 was prepared in first by modifying PF127 with homofunctionalized diethylene glycol moiety using CDI reagent followed by decoration of FA using zero length cross linkers (EDC and NHS). The brief schematic representation of synthesis is shown in Fig. 1. Fe_3_O_4_ synthesis was brought about by precipitating iron (II) and iron (III) by ammonia. Yellow-colored iron salts’ solution changed to black upon addition of ammonia. Black-colored nanocrystals thus formed were coated with oleic acid. Modification with oleic acid leads to greasy aggregations. Furthermore, the addition of Pluronic F127 and Pluronic F127 FA brought about good dispersity and solubility to the composite. The N_2_ atmosphere was maintained throughout the reaction to evade oxidation of nanoparticles. The nanocomposite centers iron oxide with subsequent hydrophobic and hydrophilic compartments. The nanocomposites, PTX-CUR-OAMNPPF127FA, were prepared, as shown in the graphical abstract.

### Fourier transform infrared spectra

**Fig. 2:**
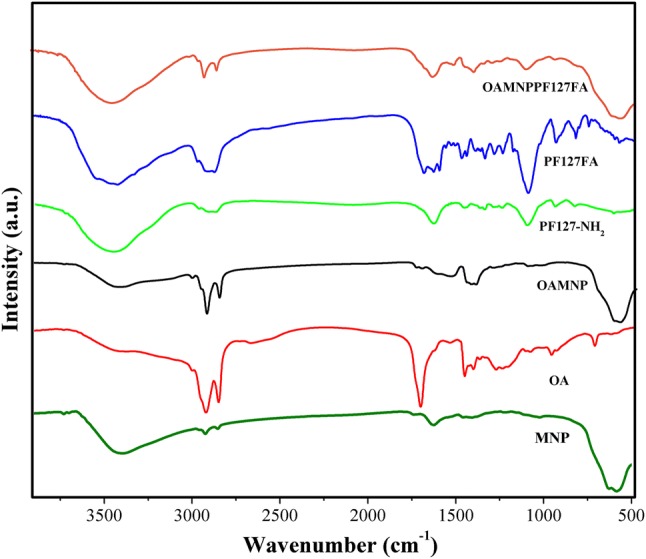
Fourier transform infrared spectra of MNP, OA, OAMNP, PF127NH_2_, PF127FA, and OAMNPPF127FA

FTIR spectra of Pure OA, MNP, OAMNP, PF127-NH2, PF127FA, and OAMNPPF127FA are shown in Fig. 2. FTIR spectra of pure OA express prominent peak at 1705/cm credible to the C = O stretch. 3000/cm broad peak is due to the –OH stretch. 2853/cm and 2922/cm peaks correspond to the CH_2_ symmetric and CH_2_ asymmetric stretching, respectively. Spectra of OAMNP, however, display no peaks corresponding to C=O stretch. The peaks at 2853/cm and 2921/cm are owed to CH_2_ groups of chemisorbed OA. The presence of peak at 1110.42/cm in PF127-NH_2_ sample corresponds to C–N stretching. Spectra of OAMNPPF127FA present peaks around 3450/cm is due to NH_2_ groups, which are merged with hydroxyl peaks. Peaks at 1693/cm and 1638/cm are due to carbonyl stretching peaks of folic acid. Peaks at 1606/cm and 1577/cm are due to a *p*-aminobenzoyl-l-glutamic acid moiety of folic acid. Spectra of OAMNPPF127FA show peaks that are in PF127FA and OAMNP.

### NMR

**Fig. 3:**
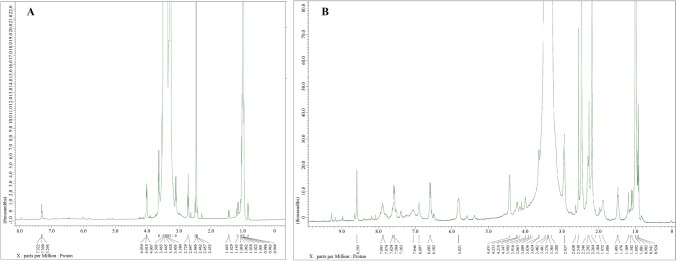
NMR spectra of PF127-NH_2_ (**a**) and PF127-FA (**b**)

1H NMR spectrum (400 MHz, DMSO-D6) of PF127-NH2 illustrated the peaks at d (ppm) = 1.02–0.95 (d, 3H × 65, –CH_3_ of PPO), 3.61–3.26 (m, 3H × 65, 4H × 200, 2H × 12, –CH_2_CHO of PPO, –CH_2_CH_2_O– and –OCH_2_CH_2_O– of PEO), 2.73 (t, 2H × 2, –CH_2_N), 3.19 (q, 2H × 2, CNCH_2_–) (Fig. 3a).

1H-NMR (400 MHz, DMSO-D6)of PF127FA 1.09 (d, 3H × 65, –CH_3_ of PPO), 2.63 (t, 2H × 2, –CH_2_N), 2.93 (m, 2H, C22 × H2 of folic acid), 3.28 (q, 2H × 2, CNCH_2_–), 3.29–3.62 (m, 3H × 65, 4H × 200, 2H × 12, –CH_2_CHO of PPO, –CH_2_CH_2_O– and –OCH_2_CH_2_O– of PEO), 4.43 (d, 2H, C9 × H2 of folic acid), 6.60 (d, 2H, aromatic protons of folic acid), 7.56–7.58 (d, 2H, aromatic protons of folic acid), and 8.59 (s, 1H, C7 × H of folic acid) (Fig. 3b).

### UV spectra

**Fig. 4:**
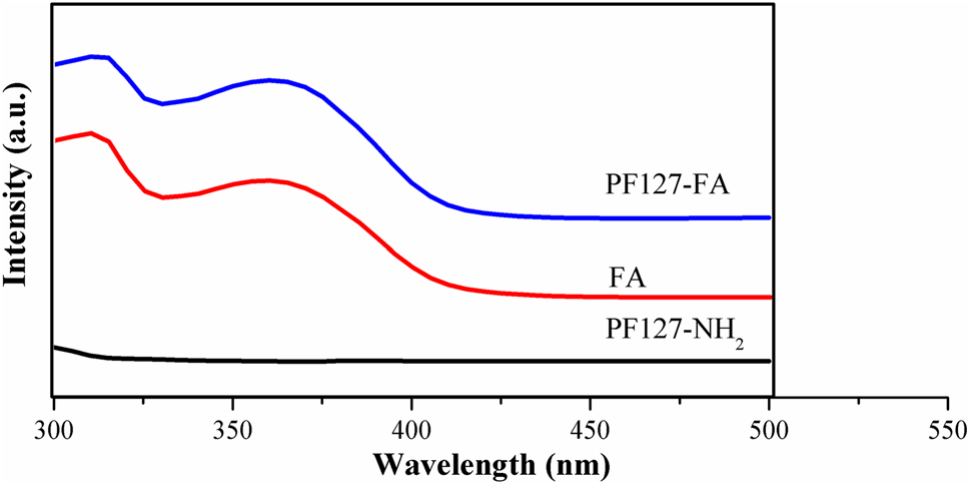
UV–visible spectra of PF127-NH_2_, FA, and PF127-NH_2_

UV–visible spectra scanned in the range of 300–500 nm of P127-NH2, FA, and P127-FA are shown in Fig. 4. No prominent peaks were noticed in P127-NH_2_ spectra. The prominent peak at 320 nm and 380 nm is seen in FA sample. Similar peaks were seen in the case of P127-FA. Furthermore, the percentage of conjugation was determined using standard curve, and was found to be 84.34%.

### Particle size and zeta potential

**Fig. 5:**
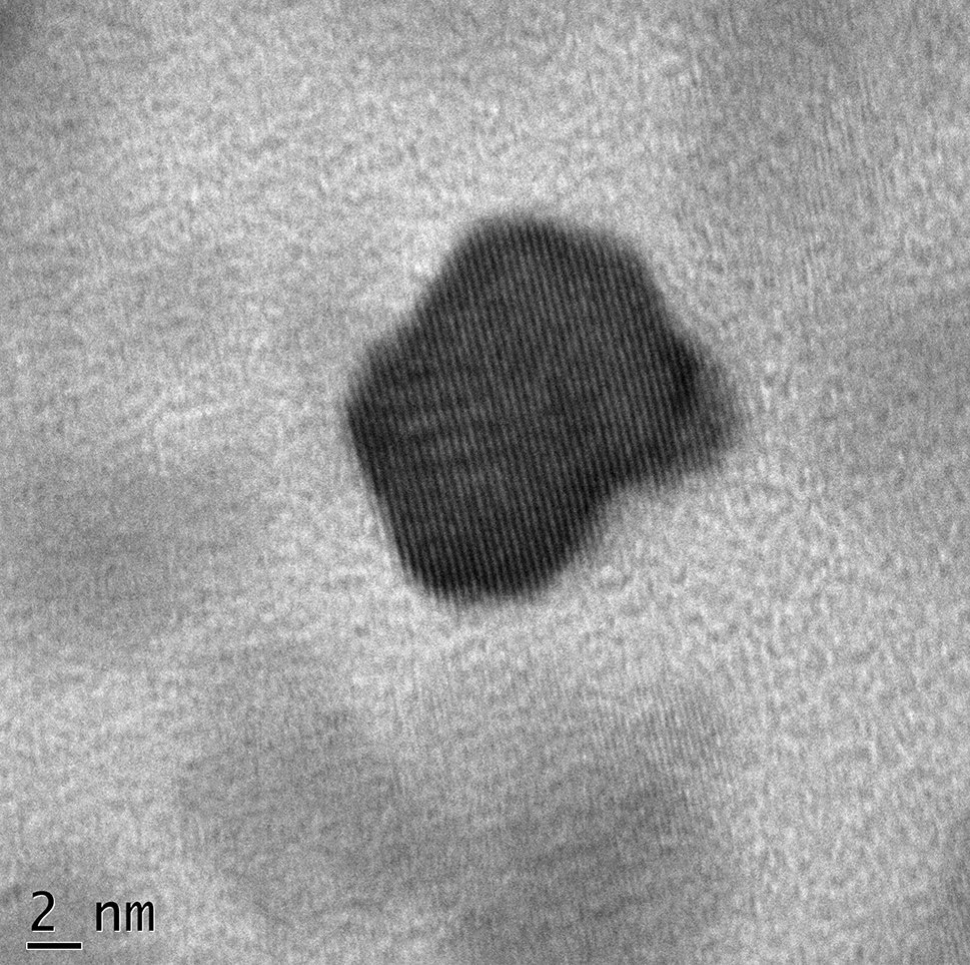
Transmission electron micrograph of OAMNPPF127FA

The hydrodynamic size, zeta potential, and polydispersity index of MNP and OAMNPPF127FA are tabulated in Table 1. MNP sample shows higher size and polydispersity index than OAMNPPF127FA. Sample OAMNPPF127FA showed much higher zeta potential in contrast to MNP. Transmission electron microscopy, which measures the size of the magnetic core, measure average particle size as 12.5 nm (Fig. 5).

**Table 1:** Size, zeta potential, and polydispersity index of MNP and OAMNPPF127FA

Sample	Size (nm)	Zeta potential (mV)	Polydispersity index
MNP	124.2 ± 9.2	− 2.1	2.313
OAMNPPF127FA	94.2 ± 6.3	− 25.5	1.011

### X-ray diffraction

**Fig. 6:**
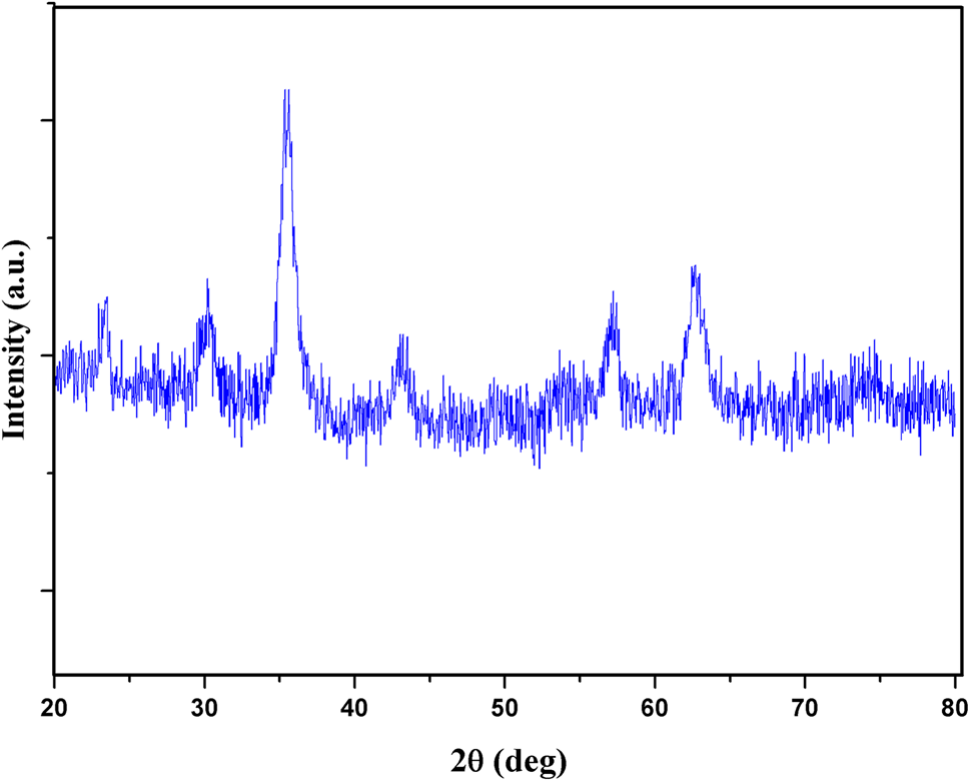
X-ray powder diffraction pattern of OAMNPPF127FA

The study of X-ray powder diffraction (XRD) was carried out to confirm the presence of nanocrystalline and the phase of the synthesized materials. The recorded XRD pattern is shown in Fig. 6. Here, we observed strong and sharp peaks, which reveal that the synthesized nanoparticles are crystallized in nature. The Bragg’s reflection angles were obtained at 2*θ* = 30.09 °C, 35.44 °C, 37.07 °C, 43.07 °C, 56.96 °C, 62.55 °C, and 74.0 °C corresponding to (220), (311), (222), (400), (511), (440), and (533) planes which are compared with the standard joint committee on powder diffraction standards (JCPDS) data and it is well matched with data no. 870245. It is confirmed that the material is iron oxide Fe_3_O_4_ having the face-centered-cubic structure of Fe_3_O_4_. Some of the observed sharp and intense peaks suggest the strong X-ray scattering centers in the crystalline phase.

### Thermogravimetric analysis (TGA)

**Fig. 7:**
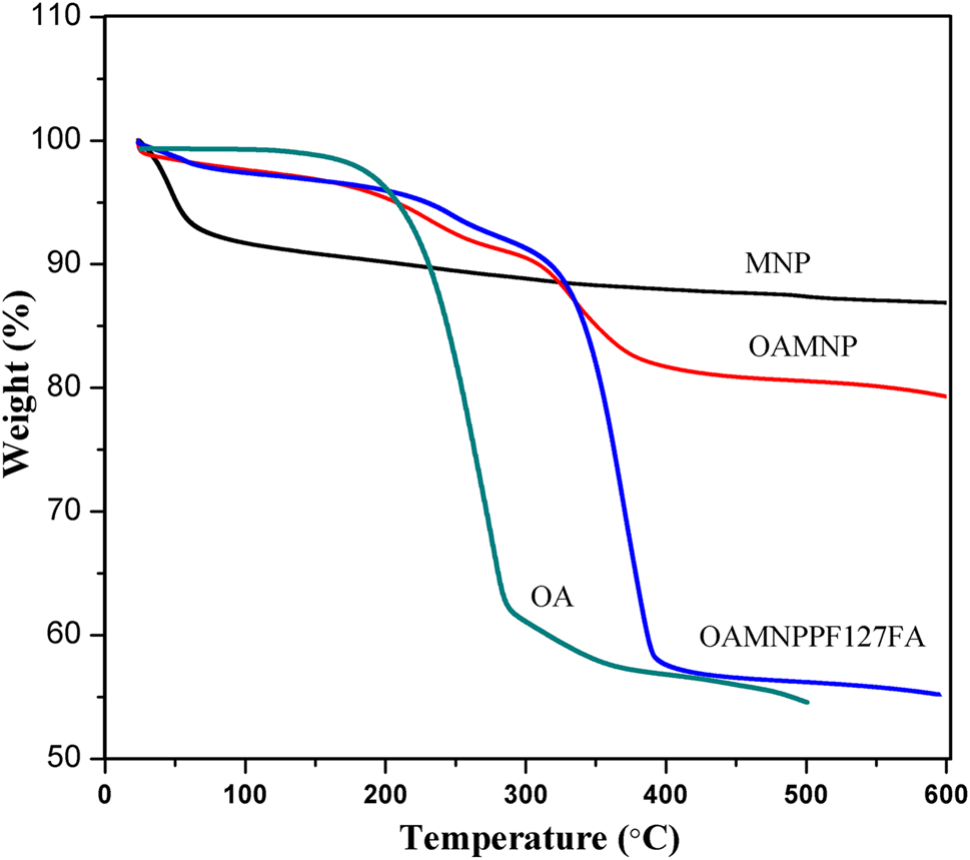
Thermogravimetric analysis of pure OA, MNP, OAMNP, and OAMNPP127FA

Thermogravimetric analysis results of pure OA, MNP, OAMNP, and OAMNPP127FA are shown in Fig. 7. Pure OA started decomposing at 200 °C, whereas the OAMNP shows steady mass around 350 °C. OAMNP and OAMNPPF127 showed an overall loss of 20.2% and 44.5%, respectively. Furthermore, mass loss witnessed at around 100 °C for MNP and OAMNPP127FA sample was not prominent. However, in the case of OAMNP, no mass loss was seen at around 100 °C.

### Vibrating sample magnetometry (VSM)

**Fig. 8:**
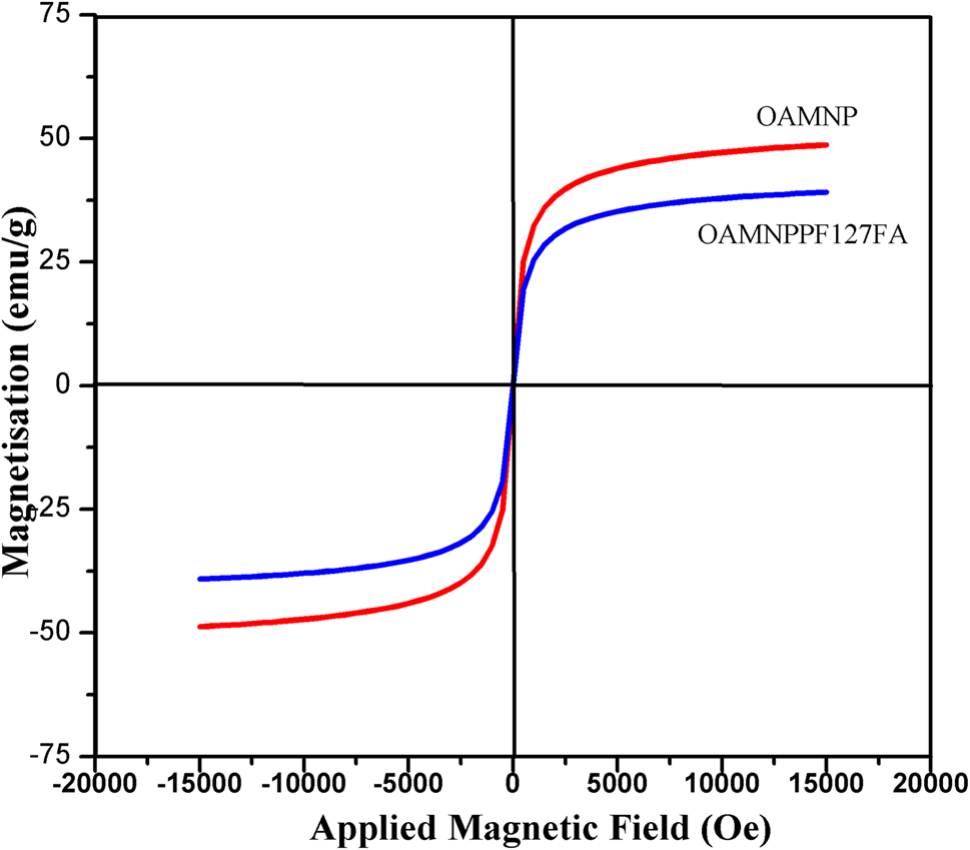
Magnetic hysteresis analysis of OAMNP and OAMNPP127FA

Magnetization and demagnetization curve of OAMNP and OAMNPP127FA samples are shown in Fig. 8. Magnetic saturation (*M*_s_) value of 49.56 emu/g and 39.04 emu/g was exhibited by OAMNP and OAMNPP127FA, respectively. Neither OAMNP nor OAMNPP127FA showed coercivity or magnetic remanence.

### Drug entrapment efficiency and in-vitro drug release

**Fig. 9:**
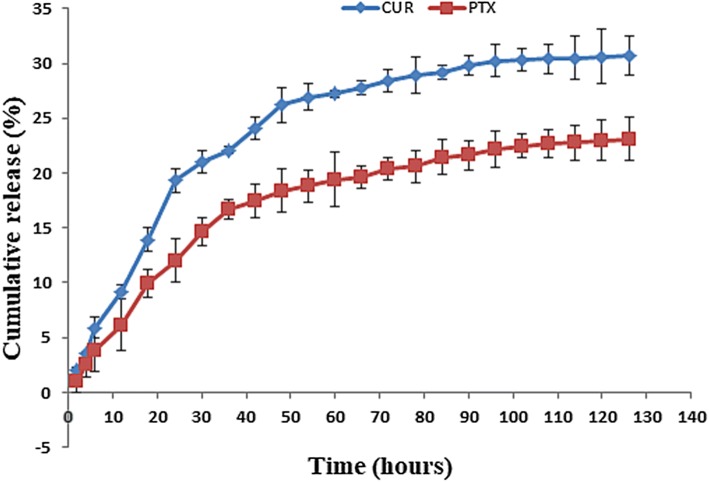
PTX and CUR release profile from OAMNPPPF127FA

Nanocomposites PTX-CUR-OAMNPPF127 had drug content of 9.42%, of which 3.47% was paclitaxel and 5.95% was curcumin. Encapsulation efficiency of paclitaxel and curcumin was 34.7 and 59.5, respectively. Furthermore, both drugs displayed sustained release behavior (Fig. 9) over 75 h.

### Hemolysis assay

**Fig. 10:**
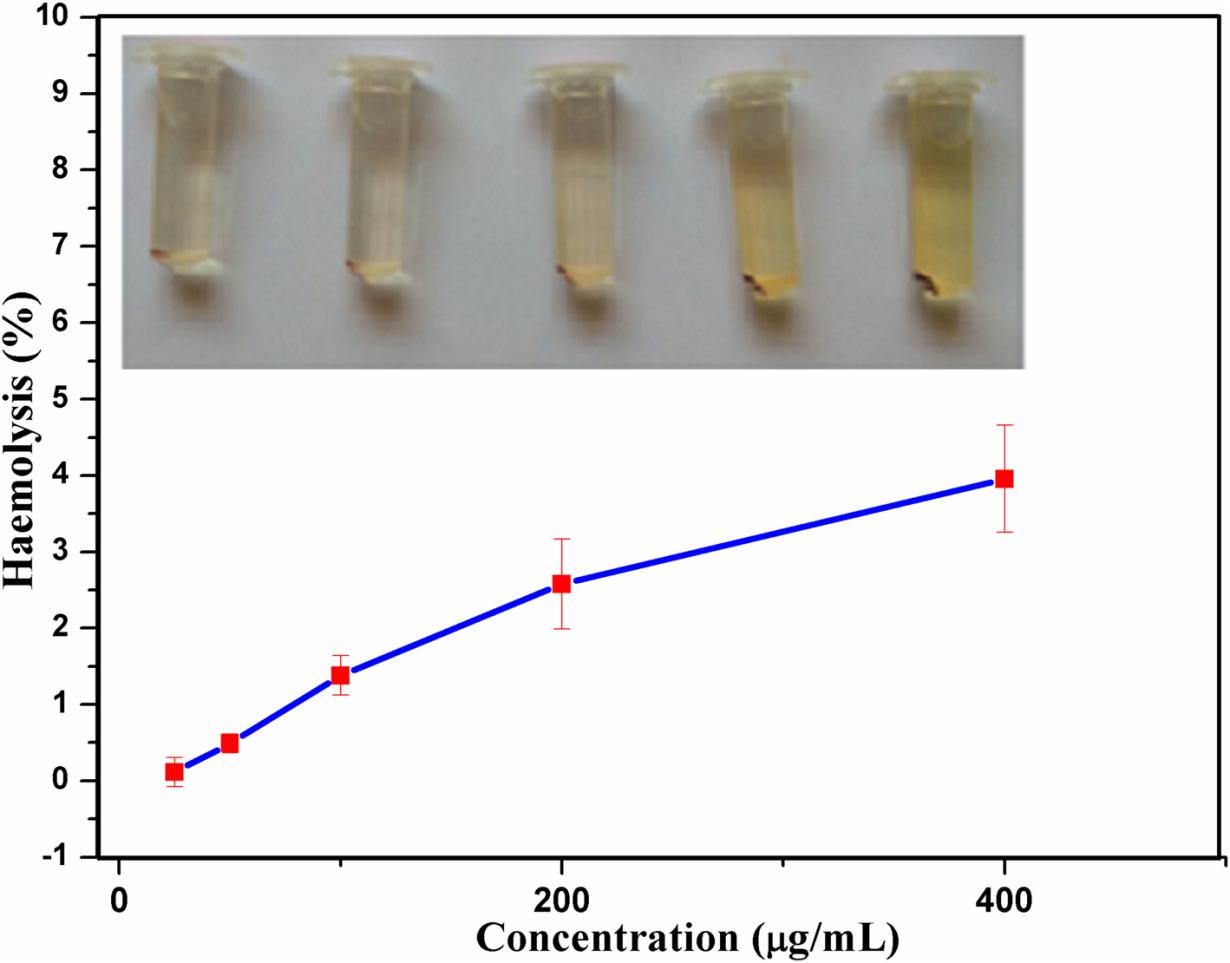
Haemolysis assay of OAMNPPF127FA at various concentrations

Haemocompatibility was measured by hemolysis assay. The amount of hemoglobin released from blood cells after incubation was determined photometrically. Figure 10 shows hemolytic activity exhibited by OAMNPP127FA nanocomposites at various concentrations. Concentration measured by UV–visible spectrophotometer at 521 nm indicated a total of 4.1% hemolysis at 400 mg/mL concentration.

### In-vitro cytotoxicity of PTX-CUR-OAMNPP127FA

**Fig. 11:**
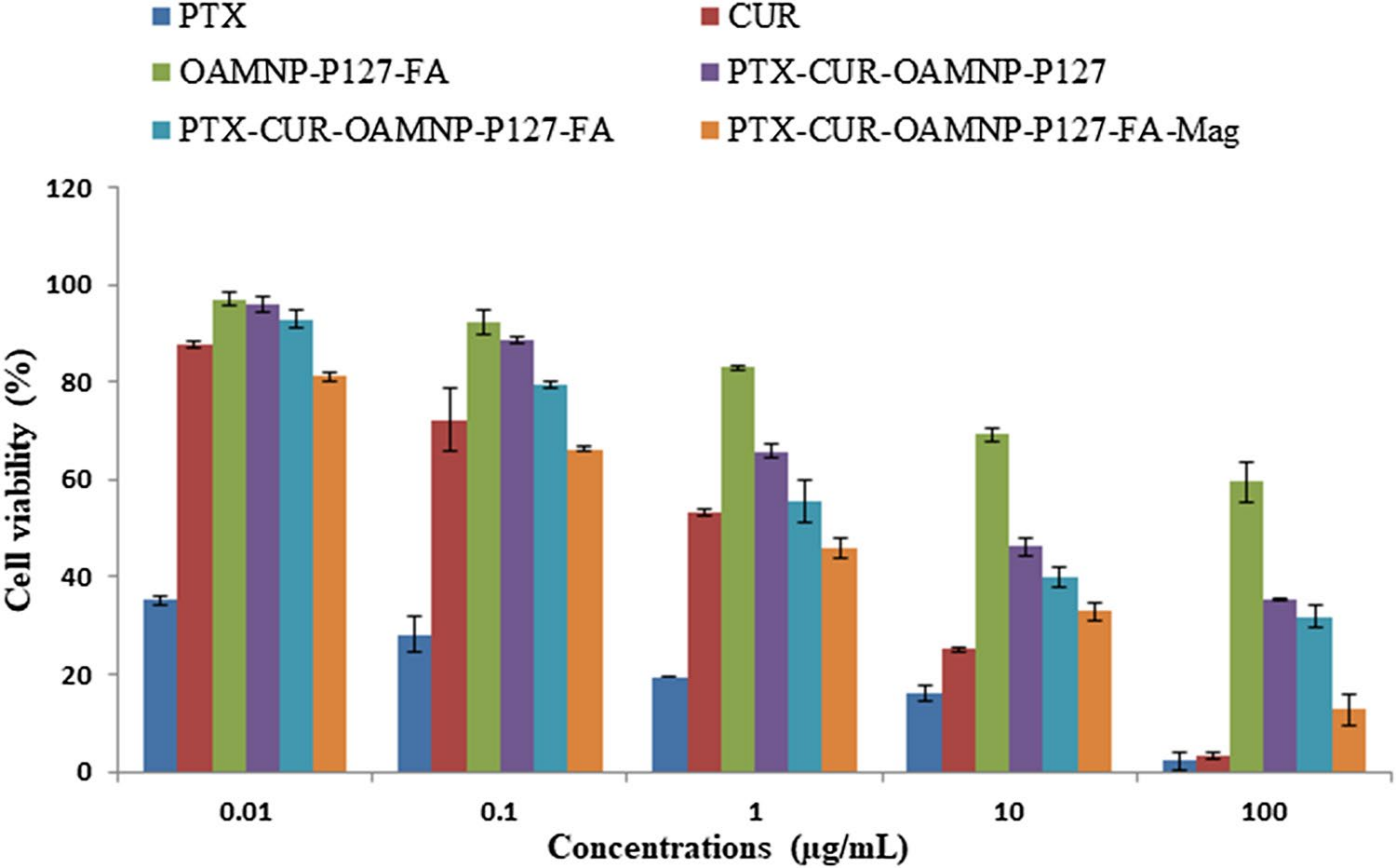
Cell viability assay of PTX, CUR, OAMNPPF127FA, PTX-CUR-OAMNPPF127FA, and PTX-CUR-OAMNPPF127FA under magnetic field at against MCF-7 cells

MTT cell viability assay was performed for 48 hr to evaluate the cytotoxic property. Samples PTX, CUR, OAMNPPF127FA, PTX-CUR-OAMNPPF127FA, and PTX-CUR-OAMNPPF127FA under magnetic field at against MCF-7 cell lines and the results thus obtained are presented in Fig. 11. The determined IC_50_ values of the PTX, CUR, Blank OAMNPPF127FA, PTX-CUR-OAMNPPF127, PTX-CUR-OAMNPPF127FA, and PTX-CUR-OAMNPPF127FA above magnetic field for 48 h were 0.002, 0.9101, 1198.67, 9.99, 4.12, and 0.7377 µg/mL, respectively.

### Nanoparticle uptake by MCF-7 cells

**Fig. 12:**
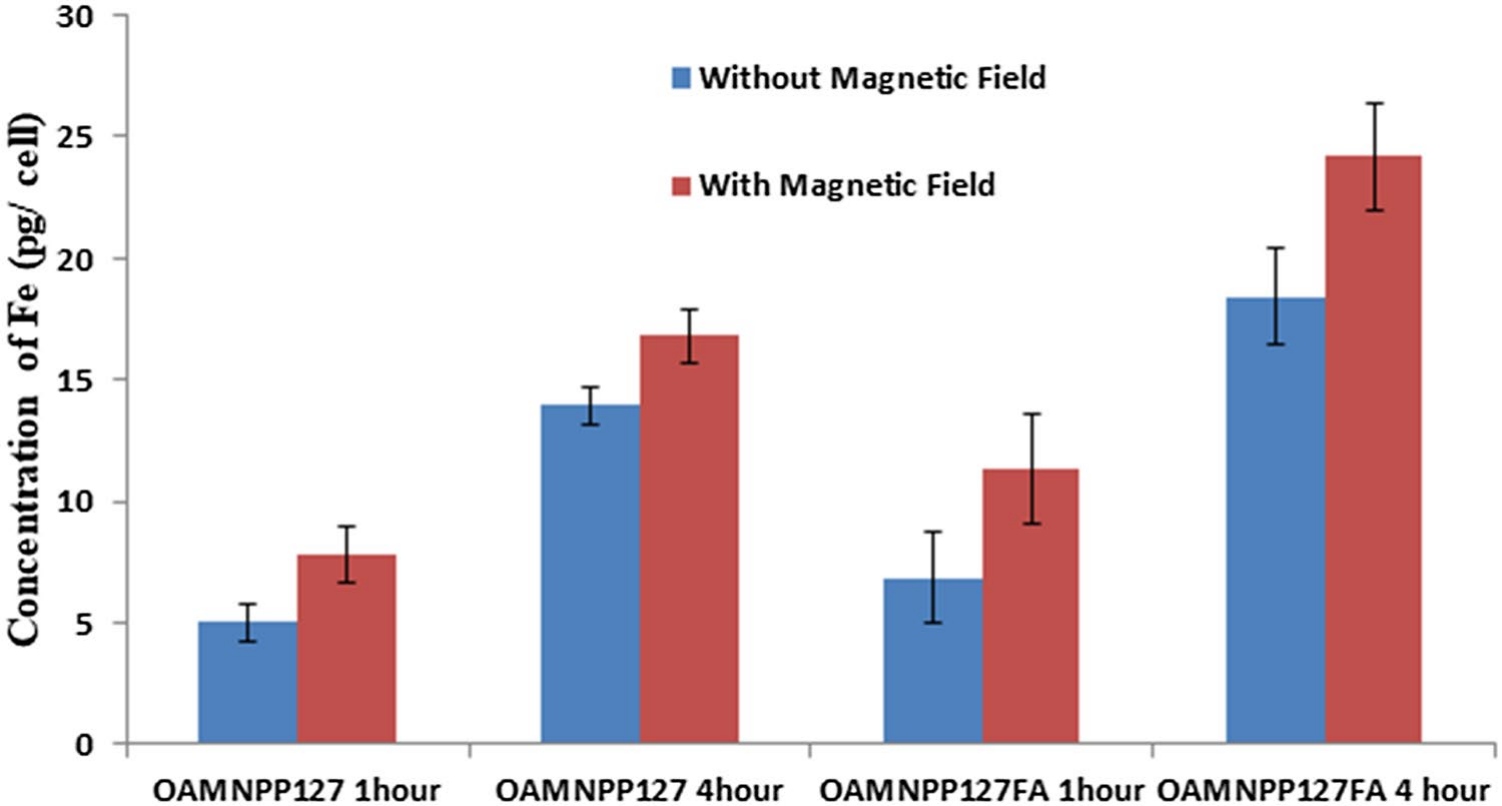
Cellular uptake of OAMNPPF127 and OAMNPPF127FA at 1 and 4 h incubation

Quantitative nanoparticle uptake was determined by assessing iron content in the cell by AAS is depicted in Fig. 12. OAMNPPF127 showed the iron concentration of 5.02 pg/cell and 13.95 pg/cell in the absence of magnetic field at 1 and 4 h of incubation. Similarly, 7.777 pg/cell and 16.8 pg/cell concentration of iron was analyzed at 1 h and 4 h incubation, respectively, in the presence of a magnetic field. OAMNPPF127FA sample displayed the iron concentration of 6.874 pg/cell and 18.4 pg/cell at 1 h and 4 h incubation in the absence of magnetic field. The presence of magnetic field and nanocomposites under magnetic field at 1 h incubation revealed the iron concentration of 2.78 pg/cell and 4.19 pg/cell in occurrence of an exterior magnetic field. At 4 h incubation, iron content of 12.31 pg/cell and 21.2 pg/cell was noticed in the absence and occurrence of a magnetic field, respectively.

## Discussion

In this work, we have synthesized OAMNPPF127FA, wherein iron oxide nanoparticle core is stabilized by OA and further coated with PF127FA and PF127 in a specific ratio. OAMNPPF127FA is a water dispersible nanoparticle-based formulation to address the often faced problem of aqueous insolubility and systemic toxicity. Lack of aqueous solubility hinders the bioavailability thus extensively hampering the effectiveness of the drug. Diethylene glycol bis(3-aminopropyl) ether is a diethylene glycol derivative and is not reported previously in the literature. Magnetic nanoparticles can be passively targeted with the external magnetic field to the site of cancer, which increases the concentration at the site of action (Pankhurst et al. 2003). We have thus constructed a nanostructure that can transport hydrophobic drugs and has the ability to concentrate to a specific area by the external magnetic field and actively targeted with the help of folic acid ligand. Diethylene glycol bis(3-aminopropyl) ether, a diethylene glycol derivative, has not been used previously for amine functionalization of PF127. We theorize that the formulation allows partitioning hydrophobic drug in the hydrophobic case neighbouring iron oxide nanoparticles.

In FTIR spectra, the presence of peaks at 2853/cm and 2922/cm indicates the presence of OA on MNP. The presence of C=O peak in OA, but absence in OAMNP confirmed the chemisorption of OA (Raut et al. 2010). The 1110.42/cm peak corresponds to C–N starching, indicating the presence of the amine group. Conjugation of folic acid to PF127FA was confirmed by peaks 1693/cm and 1638/cm, which are due to carboxyl groups of FA and peaks at 1606/cm and 1577/cm are due top-aminobenzoyl-l-glutamic acid moiety of folic acid, demonstrating conjugation of FA to PF127-NH_2_ (Li et al. 2012; Hiremath et al. 2018).

NMR spectra further confirm the conjugation of diethylene glycol bis(3-aminopropyl) ether in PF127-NH_2_ and folic acid to PF127-NH_2_. UV–visible spectra of PF127-NH_2_ showed no peaks. However, peaks similar to peaks of FA were seen in PF127FA sample, indicating successful conjugation of FA to PF127-NH_2_ (Zhang et al. 2011). Further quantitatively analysis revealed ~ 85% of conjugation.

The hydrodynamic size of MNP was found to be bigger than OAMNPPF127FA. High polydispersity index profile of MNP indicates large size distribution. This may be is due to the agglomeration of particles. The agglomeration might be owing to the absence of capping agent and smaller zeta potential (Lu et al. 2007). Zeta potential is a net superficial charge on the on nanoparticle, which is an indication degree of repulsion between particles in of similar charge in suspension. Thus, greater the negative or positive value of zeta potential, lesser the aggregation behavior of the particles (Arya et al. 2011). OAMNPPF127FA displayed good valve of zeta potential and low polydispersity index and, however, showed zeta potential of − 25.5 mV and indicating good aqueous stability. Low polydispersity index indicates a narrow size distribution profile hence lack of agglomerations. TEM images show the average particle diameter size of 12.54 nm. The obvious difference is due to coating and hydrodynamic size.

Percentage of components was revealed by thermogravimetric analysis. Iron oxide core was the major constituent making up ~ 55.5% weight of nanocomposites. Unlike pure OA decomposition curve, OAMNP decomposition curve displayed study decomposition. This can be accredited to due to chemisorption of oleic acid which might have conferred the thermal stability to OA.

The thermogravimetric analysis indicates that around 20.2% of OAMNP is OA. In the case of OAMNPPF127, approximately 53% weight loss was observed which indicate that 23% of total mass is coated with PF127 and PF127FA. Not much of weight loss noticed in case of pure MNP, and slight weight loss can be accredited to chemisorbed moisture. OAMNPPF127FA and MNP samples exhibited mass loss; however, no moisture loss was observed by OAMNP. This may be due to the hydrophobic property of OAMNP, wherein carboxyl group is chemisorbed exposing hydrophobic tail, thus repelling water.

High saturation magnetization value and super paramagnetism are of vital importance for magnetic targeting; thus, VSM analysis was performed. Super paramagnetism is a phenomenon, where nanoparticles are magnetized and completely demagnetized in the presence and absence of magnetic field, respectively. Super-paramagnetic behavior is typical for magnetic nanoparticles, since the demagnetization temperature will be less than or equal to room temperature (Wahajuddin 2012). A drop in saturation magnetization value after the coating is due to the change in iron oxide core and coating ratio (Kolhatkar et al. 2013). OAMNP and OAMNPP127FA samples exhibit magnetisation and demagnetization devoid of remanence and coercivity and thus exhibit super paramagnetism property.

Efficient drug uptake and controlled release behavior are of chief importance for a drug-delivery system (Din et al. 2017). Controlled release behavior is effective in maintaining active plasma or cancer microenvironment drug concentration (Wahajuddin 2012). Good encapsulation efficiency of 37.9% and 56.5% was exhibited by paclitaxel and curcumin, respectively. Furthermore, both drugs elicited sustained release profile (Fig. 8) over 75 h. Demonstrating OAMNPPF127FA would be excellent vehicles for hydrophobic drug loading. The in vivo applications of the biomaterial must essentially promise excellent blood compatibility (Brash 2018), such as a very low hemolytic consequence. Nanocomposites intended for biomedical application was subjected to haemolysis assay to analyze haemocompatibility (Wu et al. 2011). OAMNPPF127FA nanocomposites showed good haemocompatibility at a concentration as high as 400 µl/mL with 3.5% hemolysis.

MTT assay was used to assess the cytotoxicity of nanoparticles. All the samples showed increased cytotoxicity with an increase in concentration. Although OAMNPPF127FA exhibited some degree of cytotoxicity, the toxicity of drug-loaded nanoparticles were much higher. This is mainly accredited to the presence of the drug in the nanoparticles. Cells were treated with PTX-CUR-OAMNPPF127 in the presence of magnetic field showed relatively lower IC_50_ values in contrast to the absence of a magnetic field. This can be owed to the relatively high concentration of nanoparticles at the exterior of the cell. Resulting high number of nanocomposites at proximity and eliciting higher toxicity (Plank et al. 2003). Lesser IC_50_ in presence of magnetic field can also attributed to higher uptake of cells by nanoparticles.

Cell uptake assay was performed to analyze the uptake of nanoparticles by MCF-7 cells. Folic acid is reported to efficiently internalize folic acid and folic acid conjugates by receptor-mediated endocytosis, and its efficiency is owed to higher of Kd ~ 10^−10^M (Liu et al. 2009). Results revealed that cellular uptake increased with incubation time for all the composites. Cells incubated with FA-conjugated nanocomposites displayed higher iron concentration in comparison with unconjugated nanocomposites, thus indicating the role of FA in cellular internalization of nanocomposites. Furthermore, still, higher uptake was witnessed in the presence of magnetic field indicating a positive consequence of the external magnetic field on cellular internalization.

## Conclusions

The engineered nanoparticle can be prepared and loaded with hydrophobic agents, in this case, PTX and CUR. Since drug loading is due to the interaction of the hydrophobic cavity with the hydrophobic drug, it can be used with other hydrophobic drugs. PF127 and PF127FA have brought about good dispersity and optimum hydrodynamic size. Diethylene glycol bis(3-aminopropyl) ether can be used as a spacer for folic acid conjugation. The nanocomposites showed good encapsulation efficiency and release kinetics. The folic acid conjugated sample showed better cytotoxicity and higher cellular uptake. Nanocomposites showed that super-paramagnetic activity hence can be used for magnetic targeting. Further increase in cytotoxicity and cellular uptake was noticed in occurrence of external magnetic field. Nanocomposites showed good haemocompatibility and hence can be used for intravenous administration. We can anticipate the combination of multiple drugs and folic acid targeting could be employed for MDR cancer treatment.

## Electronic supplementary material

Below is the link to the electronic supplementary material.

Supplementary material 1 (PDF 674 kb)
