# Supplementary material for: Co-delivery of paclitaxel and curcumin to foliate positive cancer cells using Pluronic-coated iron oxide nanoparticles
Source: Prog Biomater. 2019 Jun 13;8:155–68. doi: 10.1007/s40204-019-0118-5 (PMC6825627; doi:10.1007/s40204-019-0118-5)
Supplement: Supplementary file 1 — Supplementary material 1 (PDF 674 kb) [file 40204_2019_118_MOESM1_ESM.pdf]

Standard Folic Acid Curve

Concentration(µg/mL)	Abosorbance
10	0
20	0.149
30	0.305
40	0.479
50	0.636
60	0.803
70	0.96
80	1.121
90	1.264
100	1.419

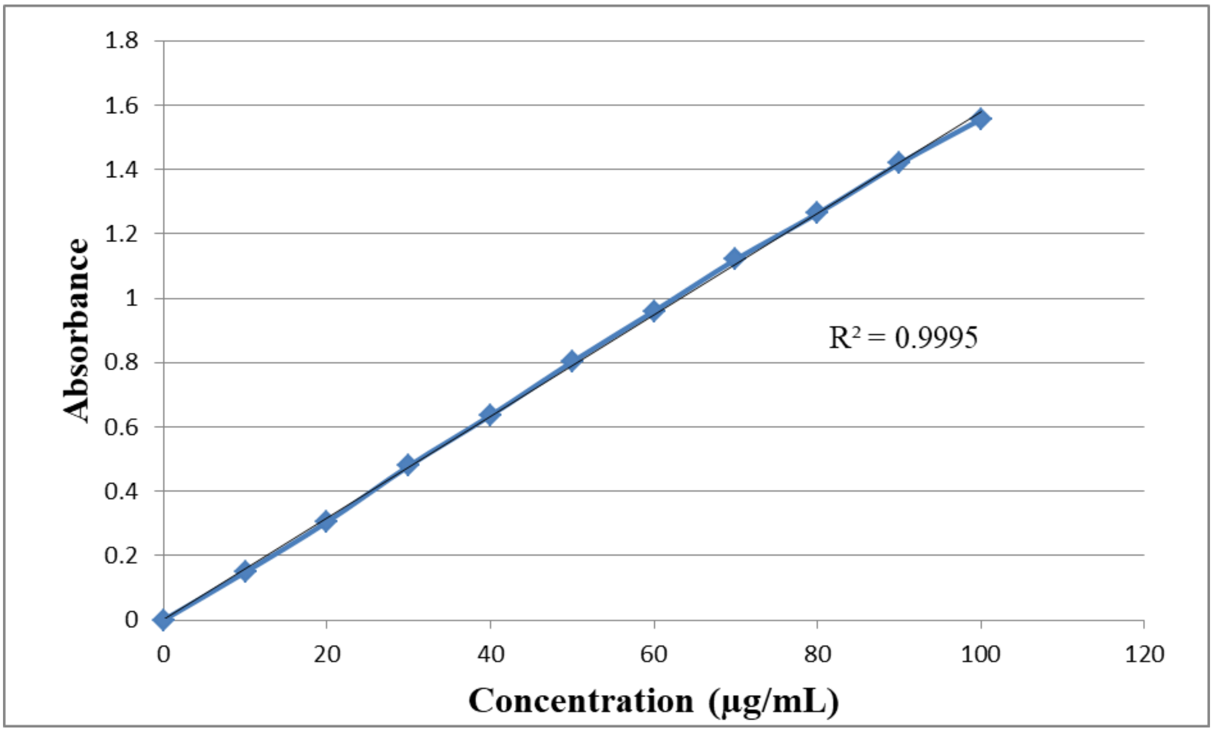

Particle size, zetapotential and Polydispersity index of MNP and OAMNPPF127FA

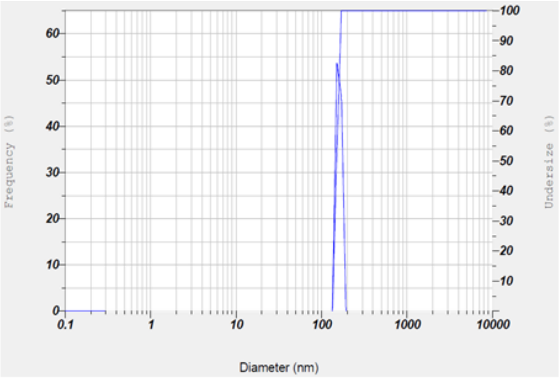

MNP Size

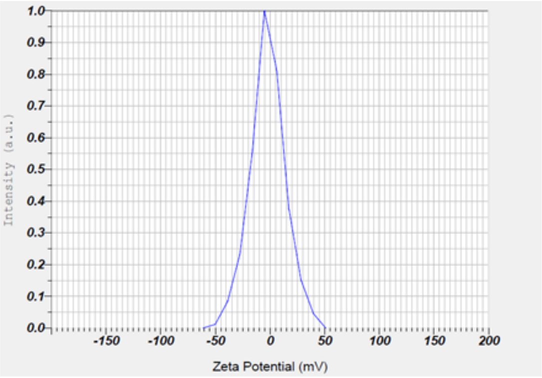

MNP Zetapotential

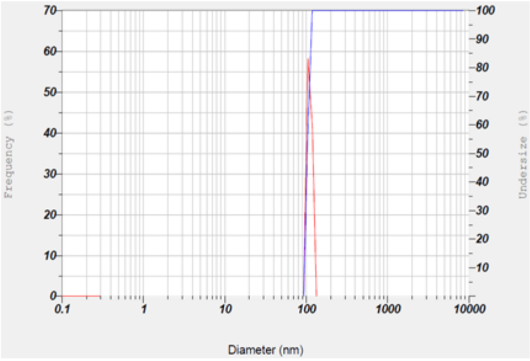

OAMNPPF127FA Size

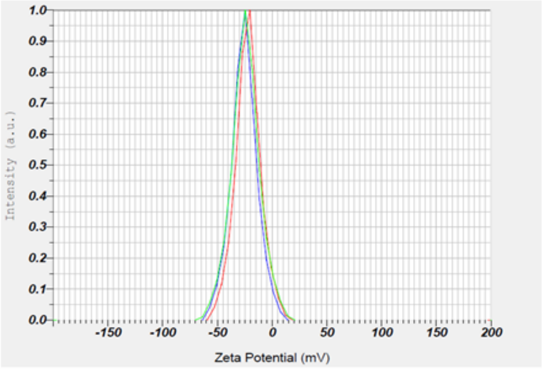

OAMNPPF127FA Zetapotential

MTT assay

Microscopic images of MTT assay at 1µg/ml concentration at 48 hours incubation.

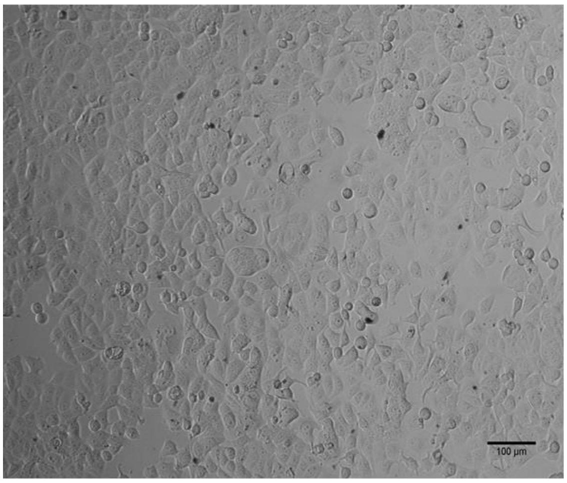

OAMNPPF127FA

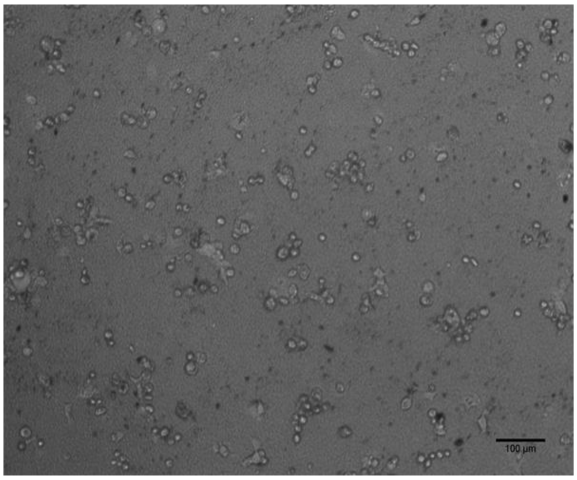

PTX-CUR-OAMNPPF127

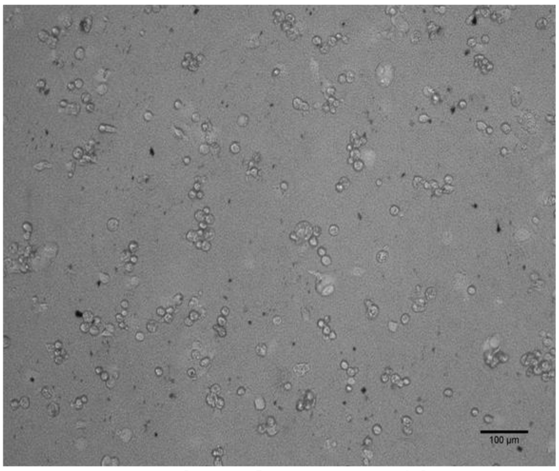

PTX-CUR-OAMNPPF127FA

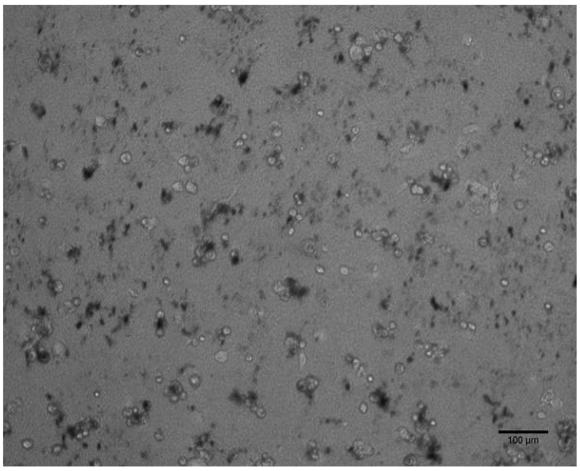

PTX-CUR-OAMNPPF127FA- Magnetic Field

Cell Uptake

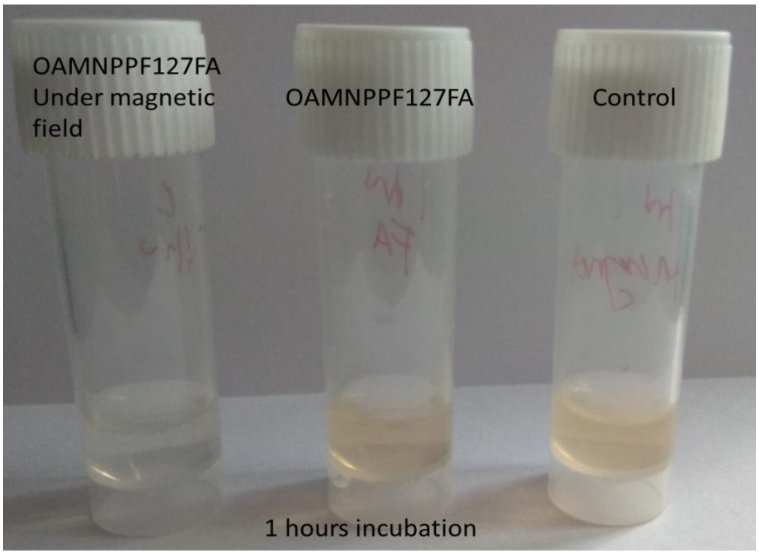
OAMNPPF127FA
Under magnetic
field
OAMNPPF127FA
Control
1 hours incubation

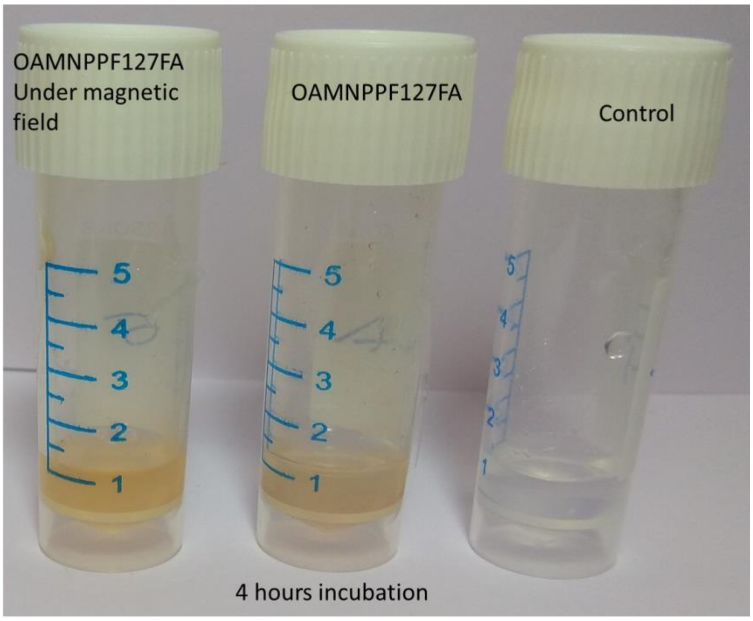
OAMNPPF127FA
Under magnetic
field
OAMNPPF127FA
Control
4 hours incubation
